# Relation between admission plasma fibrinogen levels and mortality in Chinese patients with coronary artery disease

**DOI:** 10.1038/srep30506

**Published:** 2016-07-26

**Authors:** Yong Peng, Hua Wang, Yi-ming Li, Bao-tao Huang, Fang-yang Huang, Tian-li Xia, Hua Chai, Peng-ju Wang, Wei Liu, Chen Zhang, Mao Chen, De-jia Huang

**Affiliations:** 1 Department of Cardiology, West China Hospital, Sichuan University, Chengdu, China

## Abstract

Fibrinogen (Fib) was considered to be a potential risk factor for the prognosis of patients with coronary artery disease (CAD), but there was lack of the evidence from Chinese contemporary population. 3020 consecutive patients with CAD confirmed by coronary angiography were enrolled and were grouped into 2 categories by the optimal Fib cut-off value (3.17 g/L) for all-cause mortality prediction. The end points were all-cause mortality and cardiac mortality. Cumulative survival curves showed that the risk of all-cause mortality was significantly higher in patients with Fib ≥3.17 g/L compared to those with Fib <3.17 g/L (mortality rate, 11.5% vs. 5.7%, *p* < 0.001); and cardiovascular mortality obtained results similar to those mentioned above (cardiac mortality rate, 5.9% vs. 3.6%, *p* = 0.002). Subgroup analysis showed that elevated Fib levels were predictive for the risk of all-cause mortality in the subgroups according to age, medical history, and diagnosis. COX multivariate regression analysis showed that plasma Fib levels remained independently associated with all-cause mortality after adjustment for multiple cardiovascular risk factors (all-cause mortality, HR 2.01, CI 1.51–2.68, *p* < 0.001). This study has found that Fib levels were independently associated with the mortality risk in Chinese CAD patients.

Fibrinogen (Fib) is a key factor in blood coagulation. The association between Fib and coronary artery disease (CAD) has long been a concern. Over the last few decades, a series of studies suggested that Fib was independently associated with the development of CAD and cardiovascular events^1^,^2^. However, these studies primarily included patients free of CAD or healthy individuals at entry to investigate the relation between Fib levels and the primary prevention of CAD. A few researches has been reported on the relation between Fib levels and the secondary prevention of CAD (i.e., the relation between Fib levels and the prognosis of patients already suffering from CAD), and the existing results remain controversial^3^,^4^,^5^. Much of the available research data were acquired prior to the wide application of interventional treatment and evidence-based medicine (EBM), and little research was based on contemporary patients. Recently, Ndrepepa *et al.* reported a study based on contemporary Western populations, including patients with CAD confirmed by coronary angiography. Their results showed that Fib was an independent correlate of mortality, but it did not provide additional prognostic information beyond that provided by traditional cardiovascular risk factors^6^. In Chinese patients, there is a lack of adequate research on the relation between Fib levels and the clinical prognosis of CAD. In the present study, we performed a retrospective analysis of single-centre registry data to investigate the association between Fib levels and the clinical prognosis in Chinese CAD patients.

## Results

A total of 3020 patients with CAD were included in the study. The average age was 64.4 ± 11.0 years, and men accounted for 79.4% of the patients. Plasma Fib levels were measured within 24 hours after admission and completed prior to coronary angiography. ROC curve analysis showed that the area under the ROC curve for Fib-predicted all-cause mortality of CAD patients was 0.607 (95% confidence interval [CI] 0.57 to 0.64); the optimal cut-off value was 3.17 g/L. The patients were divided into two groups according to their Fib levels and cut-off values. As shown by the baseline data distribution in Table 1, the clinical features showed differences between groups. Higher Fib levels corresponded to higher patient age, proportion of women, and incidence of concomitant hypertension, diabetes, and cardiac dysfunction.

**Figure 1:**
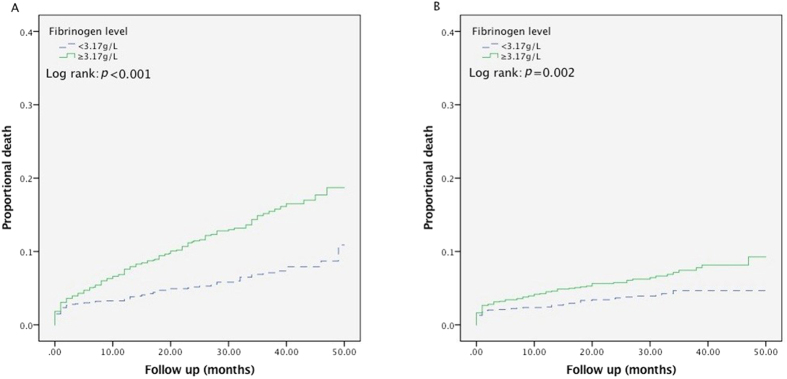
Kaplan–Meier survival curve for all-cause mortality (panel A) and cardiac mortality (panel B) in total 3020 patients with CAD according to fibrinogen levels. Abbreviations: CAD, coronary artery disease.

The 3020 patients were followed up for an average duration of 26.1 ± 13.1 months. Total of death events occurred in 258 cases (mortality rate: 8.5%) during the follow-up period, including 143 cases (cardiac mortality rate: 4.7%) of cardiac death. As shown by the cumulative survival curves of groups by Fib level, the risk of all-cause mortality was significantly higher in patients with Fib ≥3.17 g/L compared to those with Fib <3.17 g/L (mortality rate, 11.5% vs. 5.7%, *p* < 0.001) (Fig. 1, panel A). The risk analysis of cardiovascular mortality obtained results similar to those mentioned above (cardiac mortality rate, 5.9% vs. 3.6%, *p* = 0.002) (Fig. 1, panel B).

Subgroup analysis (Table 2) showed that elevated Fib levels were predictive for the risk of all-cause mortality in the subgroups according to age, medical history, and diagnosis. However, the results did not reach statistical significance in the female and renal dysfunction subgroups, possibly due to the small sample sizes of these subgroups. The interaction analysis did not reveal any interactive effects of stratified subgroups of the variables on the association between Fib levels and CAD prognosis. The results of the subgroup analysis of cardiovascular death showed trends similar to those obtained for all-cause mortality. The results did not reach statistical significance in several subgroups, possibly due to the small number of cases with cardiovascular death events and a lack of power of the test.

COX multivariate regression analysis (Table 3) showed that plasma Fib levels remained independently associated with all-cause mortality and cardiovascular mortality after adjustment for multiple cardiovascular risk factors [all-cause mortality, hazard ratio (HR) 2.01, confidence interval (CI) 1.51–2.68, *p* < 0.001; cardiovascular mortality, HR 1.58, CI 1.09–2.31, *p* = 0.016].

## Discussion

The results of this study showed that plasma Fib levels were independently associated with all-cause mortality in Chinese CAD patients. This independent association remained after adjustment for cardiovascular risk factors.

Fib, which is a glycoprotein synthesised by the liver, is a key factor in blood coagulation and a major component of thrombus. Fib is free in the plasma; the Fib concentration increases exponentially in cases of injury or inflammation and is converted to the fibrin monomer under the action of thrombin, thereby polymerizing to form a thrombus^7^,^8^. Plasma Fib has been shown to play a major role in the inflammatory response^9^, endothelial dysfunction and smooth muscle cell migration^10^. Therefore, the association of Fib levels with atherosclerosis and cardiovascular events has long been a concern^11^,^12^,^13^. Moreover, Fib levels have been shown to play an important role in atherosclerosis^14^, the severity of coronary artery lesions^15^, platelet activation and thrombosis^16^. Clinical trials based on populations free of CAD at entry suggested that Fib was significantly associated with the development of CAD and cardiovascular events^1^,^2^. However, the role of plasma Fib levels as a cardiovascular risk factor remains controversial. The Prospective Epidemiological Study of Myocardial Infarction (PRIME)^17^ and the Atherosclerosis Risk in Communities (ARIC) study^18^, which included adults without history of CAD, suggested that the independent association of Fib with the risk of cardiovascular events was abolished after adjusting for potential risk factors.

Currently, relatively few studies have reported the association between plasma Fib levels and secondary prevention of CAD. Several existing studies have come to inconsistent conclusions. Sjöland *et al.* retrospectively analysed the relation between preoperative Fib and 10-year mortality in 729 patients undergoing CABG and did not find an independent risk relation between Fib and long-term mortality^3^. Another prospective study included 719 patients with CAD diagnosed by coronary angiography who were followed up for an average duration of 6.5 years. The results showed that Fib was independently related to cardiovascular death and the extent of disease^4^. In another small sample study (n = 111) with a follow-up time of 12 years, the results showed that elevated Fib levels predicted the angiographic progression of existing coronary disease and the likelihood of cardiovascular death^5^. The AthroGene study included 1806 patients with stable angina; the results suggested that Fib was predictive for future cardiovascular risk, but the study did not provide further information beyond that obtained from models including traditional risk factors. However, the study included a portion of patients with coronary atherosclerosis who only had 30–50% stenosis of the coronary artery^19^. A recent study including 13,195 patients with CAD confirmed by coronary angiography also showed that Fib was an independent correlate of mortality but did not provide additional prognostic information on top of that provided by traditional cardiovascular risk factors^6^. To date, studies based on Chinese patients have primarily focused on the association of Fib levels with the severity of coronary artery or plasma levels of inflammatory markers in patients^20^,^21^. Few small sample studies have evaluated the prognosis^22^, and no large sample studies on the prognosis of Chinese patients with CAD have been reported. In addition to the progress of the medical level and extension of the guidelines in clinical practice, current CAD treatment has entered into an era of interventional treatment and EBMs therapy. The impact of various cardiovascular risk factors on the prognosis of patients with CAD also changes with the times. Therefore, determining whether the effect of Fib levels on contemporary patients with CAD varies is worthy of attention. In the present study, we retrospectively analysed 3020 patients with CAD diagnosed by coronary angiography who were continuously registered at a single centre. We found that elevated Fib levels were significantly associated with the mortality risk among CAD patients. This association existed independently after adjustment for multi-factors. This finding is in agreement with the result of previous studies obtained from CAD patients in Western countries [4–6], indicating that Fib levels might play a role in the secondary prevention of CAD among Chinese patients.

This study was a single-centre observational study and had a few limitations. First, the registry made it difficult to completely avoid selection bias and confounding factors. Second, the samples in this single-centre study were subject to geographical restrictions, which affected their representativeness and generalization. Finally, the observational study could only demonstrate the association between Fib levels and the prognosis of patients with CAD but could not provide conclusions for causality. In summary, caution must be taken when analysing the results of this study. We expect that prospective multi-centre studies with higher quality will provide more evidence in the future.

In conclusion, Fib levels were independently associated with the mortality risk in Chinese CAD patients, indicating that Fib levels may play a role in the secondary prevention of CAD among Chinese patients.

## Methods

### Study population

The data source for this investigation was the West China Hospital CAD database. This single center database prospectively includes all the CAD or high risk patients undergoing angiography in West China Hospital, a 4950-bed teaching hospital affiliated to Sichuan University. For this analysis, we enrolled consecutive patients with CAD from January 2009 to September 2012 of the database. Patients with CAD were eligible for inclusion if they were restricted to participants with angiographic evidence of ≥50% stenosis in ≥1 coronary vessels. In addition to the above angiographic criteria, patients with ACS were eligible for inclusion if they had the following criteria: (1) ischemic chest discomfort that increased or occurred at rest; and (2) elevated cardiac troponin I levels (≥0.03 μg/L) or elevated cardiac troponin T levels (≥42 ng/L); and/or (3) new or presumably new electrocardiographic deviation in at least two contiguous leads (either pathologic Q waves (≥0.04 s in duration), ST segment dynamic horizontal/down-sloping depression ≥0.05 mV, or persistent ST segment elevation ≥0.1 mV in ≥2 contiguous precordial leads or ≥2 adjacent limb leads or new left bundle branch block (LBBB)). The exclusion criteria included malignancies, pregnancy, end stage renal disease (ESRD) or renal transplant and severe liver or hematological diseases. These inclusion and exclusion criteria were met by 3365 continuously enrolled CAD patients. After excluding patients with loss of follow-up (n = 287) or incomplete follow-up data (n = 58), 3020 patients were included in the data analysis. The study protocol was approved by the Ethics Committee of West China Hospital affiliated to Sichuan University. The methods were carried out in accordance with the approved guidelines. All subjects provided written informed consent before enrolment.

### Baseline characteristics

Demographic data, medical history, cardiovascular risk factor, vital signs at admission, medication at discharge, and final diagnosis were obtained from the patients’ electronic medical records and reviewed by a trained study coordinator. Blood sample were collected at admission and before angiography, and plasma biomarkers including fibrinogen, liver and kidney function, blood glucose, serum lipid, etc. were analyzed in the department of Laboratory Medicine, West China hospital, accredited by the College of American Pathologists. Fibrinogen is assayed by Clauss method by the automatic coagulometer (Symex CA-7000, Japan). Hypertension was defined as those with systolic blood pressure (SBP) ≥ 140 mm Hg and/or diastolic blood pressure (DBP) ≥ 90 mm Hg and/or those receiving antihypertensive medications. Diabetes mellitus (DM) was diagnosed in patients who had previously undergone dietary treatment for diabetes, had received additional oral antidiabetic or insulin medication or had a current fasting blood glucose level of ≥7.0 mmol/L or random blood glucose level ≥11.1 mmol/L. The Chinese Modification of Diet in Renal Disease (MDRD) equation was used to estimate glomerular filtration rate (eGFR) in milliliters per minute per 1.73 m^2^ 23. Patients received care according to the usual practice; treatment was not affected by participation in this study.

### Follow-up and end points

The follow-up period ended on January 2013. Follow-up information was collected through contact with patients’ physicians, patients or their family. All data were corroborated with the hospital records. The primary end points in this study were all-cause mortality and the secondary end points were cardiovascular death, as documented in the database. Death was considered cardiac when it was caused by acute MI, significant arrhythmias, or refractory heart failure. Sudden unexpected death occurring without another explanation was included as cardiovascular death.

### Statistical analyses

We conducted the post-hoc analysis on a retrospective basis. Baseline demographics and clinical characteristics were compared among patients categorized by the admission fibrinogen levels in two groups. Continuous variables are expressed as the mean ± standard deviation (SD), and categorical variables are reported as counts and percentages. Analysis of *t* test and chi-squared tests were used to test for differences between groups for continuous and categorical variables, respectively. Receiver-operating characteristic (ROC) curve analysis was performed to determine the best fibrinogen cut-off value for the prediction of all-cause mortality while maximizing sensitivity and specificity. All patients were grouped into 2 categories by the optimal Fib cut-off value. Kaplan-Meier survival curve of the two fibrinogen groups in relation to all-cause mortality and cardiac mortality in CAD patients was constructed and examined using the log-rank test for comparison. To assess the potential heterogeneity of the effect of admission plasma Fib levels on all-cause and cardiac mortality we performed stratification analyses. The factors of stratification included age groups (cut-off 65 years), gender, history of hypertension and DM, diagnosis of acute coronary syndrome (ACS) and eGFR (cut-off 60 ml/min). The interaction testing between fibrinogen and all-cause mortality or cardiac mortality in the patients in the variable stratifications was performed in the Cox-regression analysis. Cox proportional hazards regression models was used to investigate the independent effect of fibrinogen on all-cause and cardiac mortality. The variables included fibrinogen (grouped by the cut-off value 3.17 g/L), age (for 10-year increase), gender, history of hypertension and DM, diagnosis of ACS and eGFR (for each 30 ml/min decrease). Two-sided *p* values of less than 0.05 indicated statistical significance. All analyses were performed with SPSS software (version 19.0).

## Additional Information

**How to cite this article**: Peng, Y. *et al.* Relation between admission plasma fibrinogen levels and mortality in Chinese patients with coronary artery disease. *Sci. Rep.* **6**, 30506; doi: 10.1038/srep30506 (2016).

## Figures and Tables

**Table 1:** Baseline characteristics of the study population.

Characteristics		Fibrinogen (g/L)		*p* value
	Total	<3.17	≥3.17	
No. of patients	n = 3020	n = 1548	n = 1472	
Age, yrs	64.4 (11.0)	63.3 (11.2)	65.6 (10.5)	<0.001
Gender, men, n (%)	2399 (79.4)	1272 (82.2)	1127 (76.6)	<0.001
Medical history				
Current smoking, n (%)	990 (32.8)	536 (34.6)	454 (30.8)	0.018
Pre-hypertension, n (%)	1653 (54.7)	801 (51.7)	852 (57.9)	0.001
Pre-diabetes mellitus, n (%)	659 (21.8)	289 (18.7)	370 (25.1)	<0.001
At admission				
Systolic blood pressure, mm Hg	130.7 (22.5)	130.6 (21.7)	130.7 (23.2)	0.903
Diastolic blood pressure, mm Hg	76.5 (12.6)	76.7 (12.4)	76.3 (12.7)	0.394
Heart rate, beats/min	74.6 (23.2)	73.5 (29.1)	75.82 (14.5)	0.006
Killip classification ≥ II, n (%)	362 (12.0)	169 (10.9)	193 (13.1)	0.222
Laboratory values				
eGFR, ml/min/1.73 m^2^	97.9 (28.5)	100.6 (26.1)	95.0 (30.5)	<0.001
Blood glucose, mmol/L	7.1 (3.3)	7.0 (3.1)	7.2 (3.5)	0.031
Total cholesterol, mmol/L	4.1 (1.1)	4.6 (1.11)	4.1 (1.1)	0.270
LDL-C, mmol/L	2.5 (3.6)	2.5 (5.0)	2.4 (0.9)	0.601
Diagnosis				
ACS, n (%)	2154 (71.3)	1079 (69.7)	1075 (73.0)	0.043
Severity of CAD				
Left main artery, n (%)	283 (9.6)	138 (8.9)	145 (9.9)	0.378
Three vessel diseases, n (%)	796 (26.4)	359 (23.2)	437 (29.7)	<0.001
Discharge medication				
Aspirin, n (%)	2775 (91.9)	1428 (92.2)	1347 (91.5)	0.652
Clopidogrel, n (%)	2697 (89.3)	1365 (88.2)	1332 (90.5)	0.120
Statin, n (%)	2719 (90.0)	1401 (90.5)	1318 (89.5)	0.599
ACE inhibitors or ARBs, n (%)	1729 (57.3)	891 (57.6)	838 (56.9)	0.785
Beta-receptor blockers, n (%)	1985 (65.7)	1003 (67.8)	982 (66.7)	0.690

Data are expressed as means ± SD or counts and percentages, as appropriate.

Abbreviations: eGFR, estimated glomerular filtration rate; LDL-C, low-density lipoprotein-cholesterol; ACS, acute coronary syndrome; CAD, coronary artery disease; ACE, angiotensin-converting enzyme; ARBs, angiotensin-receptor blockers.

**Table 2:** Subgroup analyses of hazard ratio for the incidence of all-cause mortality and cardiac mortality according to the admission fibrinogen levels strata (<3.17 and ≥3.17 g/L).

Subgroups	All-Cause Mortality		Cardiac Mortality	
	HR (95% CI)	*P*_int_	HR (95% CI)	*P*_int_
All patients	2.15 (1.66–2.78)		1.70 (1.21–2.38)	
Age		0.769		0.231
≥65 years	2.90 (1.55–2.81)		1.86 (1.24–2.78)	
<65 years	1.88 (1.11–3.19)		1.17 (0.63–2.20)	
Gender		0.064		0.454
Men	2.43 (1.80–3.28)		1.80 (1.22–2.66)	
Women	1.43 (0.87–2.36)		1.33 (0.68–2.60)	
Pre-hypertension		0.061		0.586
Yes	1.72 (1.24–2.40)		1.54 (0.99–2.40)	
No	2.86 (1.88–4.33)		1.85 (1.10–3.10)	
Pre-diabetes mellitus		0.371		0.404
Yes	1.71 (1.06–2.77)		1.29 (0.68–2.44)	
No	2.24 (1.64–3.04)		1.80 (1.21–2.68)	
Renal function at admission		0.205		0.139
eGFR ≥ 60, ml/min	2.10 (1.57–2.80)		1.75 (1.19–2.56)	
eGFR < 60, ml/min	1.33 (0.74–2.80)		0.90 (0.43–1.86)	
Diagnosis		0.057		0.170
ACS	1.85 (1.39–2.46)		1.48 (1.02–2.13)	
Stable CAD	3.77 (2.04–6.95)		3.00 (1.30–6.90)	

Abbreviations: HR, hazard ratio; CI, confidence interval; *P*_int_, *p* value for interaction; eGFR, estimated glomerular filtration rate; ACS, acute coronary syndrome; CAD, coronary artery disease.

**Table 3:** Results of multivariate Cox proportional-hazards model regarding all-cause mortality and cardiac mortality.

Characteristics	All-Cause Mortality		Cardiac Mortality	
	HR (95% CI)	*p*	HR (95% CI)	*p*
Fibrinogen^*^	2.01 (1.51–2.68)	<0.001	1.58 (1.09–2.31)	0.016
Age^†^	1.54 (1.32–1.79)	<0.001	1.26 (1.04–1.53)	0.018
Men	0.95 (0.70–1.30)	0.746	1.05 (0.69–1.59)	0.820
Pre-hypertension	0.95 (0.71–1.28)	0.741	0.99 (0.67–1.48)	0.975
Pre-diabetes mellitus	1.47 (1.09–1.98)	0.011	1.42 (0.95–2.14)	0.090
eGFR at admission^‡^	1.29 (1.12–1.49)	0.001	1.36 (1.13–1.64)	0.001
LDL-C	1.02 (1.01–1.03)	<0.001	1.02 (1.01–1.03)	<0.001
Diagnosis of ACS	2.10 (1.48–2.96)	<0.001	2.65 (1.63–4.31)	<0.001
Aspirin	0.31 (0.21–0.45)	<0.001	0.30 (0.17–0.51)	<0.001
Clopidogrel	0.40 (0.27–0.60)	<0.001	0.30 (0.18–0.50)	<0.001
Statin	0.50 (0.35–0.73)	<0.001	0.50 (0.30–0.84)	0.008
ACE inhibitors or ARBs	0.72 (0.54–0.97)	0.033	0.70 (0.46–1.07)	0.096
Beta-receptor blockers	0.57 (0.43–0.76)	<0.001	0.50 (0.34–0.74)	0.001

^*^Grouped by the cut-off value 3.17 g/L; ^†^for 10-year increase; ^‡^for each 30 ml/min decrease.

Abbreviations: HR, hazard ratio; CI, confidence interval; eGFR, estimated glomerular filtration rate; LDL-C, low-density lipoprotein-cholesterol; ACS, acute coronary syndrome; ACE, angiotensin-converting enzyme; ARBs, angiotensin-receptor blockers.
